# Methanogenic Community Characteristics and Its Influencing Factors in Reservoir Sediments on the Northeastern Qinghai Plateau

**DOI:** 10.3390/biology13080615

**Published:** 2024-08-14

**Authors:** Zebi Liu, Xufeng Mao, Yi Wu, Liang Xia, Hongyan Yu, Wenjia Tang, Yanhong Qi, Ziping Zhang, Feng Xiao, Haichuan Ji

**Affiliations:** 1 Key Laboratory of Qinghai Province Physical Geography and Environmental Process, Qinghai Normal University, Xining 810008, China; liuzebi06@163.com (Z.L.); mambawu@yeah.net (Y.W.); 15079266732@163.com (L.X.); 2 Key Laboratory of Tibetan Plateau Land Surface Processes and Ecological Conservation (Ministry of Education), Qinghai Normal University, Xining 810008, China; 3 Qinghai Qilian Mountain National Park Qinghai Service Guarantee Center, Xining 810008, China; qhyuhy@163.com; 4 Qinghai Provincial Department of Ecology and Environment, Xining 810008, China; qhtsy@126.com; 5 School of Management, Wuhan University of Technology, Wuhan 430070, China; qiyanhongonline@163.com; 6 Qinghai Provincial Key Laboratory of Ecological Environment Monitoring and Assessment, Xining 810008, China; nxzhangjie@163.com; 7 Qinghai Forestry and Grass Bureau, Xining 810007, China; 13709760122@163.com (F.X.); qhsdzx@163.com (H.J.)

**Keywords:** CH_4_, Qinghai Plateau, co-occurrence network, mantel analysis, FAPROTAX

## Abstract

**Simple Summary:**

Methanogens are a group of microorganisms with the capability to convert inorganic or organic compounds into methane, impacting the global carbon cycle and greenhouse gas emissions. In anaerobic environments such as wetlands, paddy fields, and reservoirs, methanogens are the primary contributors to methane production. In this study, high-throughput sequencing of the mcrA functional gene was utilized to conduct a comprehensive analysis of the community structure, metabolic pathways, and influencing factors of methanogens in the sediment of 18 reservoirs on the northeastern Qinghai Plateau. The results indicated that the construction of reservoirs has altered the physicochemical properties of river water and sediments, significantly impacting the distribution and succession of methanogenic communities in the sediment. In general, this study can improve our understanding of the biological mechanisms of methane emissions from the reservoirs on the Qinghai Plateau.

**Abstract:**

Reservoirs are a hotspot for methane emissions, a potent greenhouse gas. However, the microbial basis for methane production in the Qinghai Plateau reservoirs remains unclear. To explore the characteristics of methanogenic communities in reservoir sediments on the northeastern Qinghai Plateau, sediment samples were collected from 18 reservoirs in the Yellow River basin during May 2023 (dry season) and August 2023 (wet season). High-throughput sequencing technology was employed to analyze the community composition, diversity, and co-occurrence network of methanogens. Furthermore, FAPROTAX and Mantel analysis were used to assess the metabolic functions of methanogens and their influencing factors. The results showed that (1) the predominant genera of methanogens were *Methanobacterium* (28.87%) and *Methanosarcina* (21.67%). Hydrogenotrophic methanogenesis was the main pathway in the sediments. (2) Significant spatiotemporal differences were observed in the diversity of methanogenic communities (*p* < 0.05). The composition and diversity of these communities were found to be significantly influenced by temperature, pH, altitude, organic carbon, and total nitrogen (*p* < 0.05). (3) *Methanosarcina*, *Methanobacterium*, and *Methanospirillum* play crucial roles in maintaining the stability of methanogenic community networks. The co-occurrence network nodes are predominantly positively correlated (99.82%). These results provide data for further studies on carbon cycling in the Qinghai Plateau reservoirs.

## 1. Introduction

As one of the significant greenhouse gasses in the atmosphere, methane (CH_4_) has a global warming potential 28 times that of carbon dioxide [1,2]. It has been confirmed that reservoirs are the major contributors to atmospheric methane [3]. A study has estimated the potential methane emissions from global reservoirs, and the results show that worldwide reservoir CH_4_ emissions amount to as high as 13.3 Tg [4,5]. China has built over 90,000 reservoirs with a total water storage capacity exceeding 8.1 × 1011 m^3^, ranking first in the world [6]. Therefore, reservoir methane emissions are becoming increasingly prominent in greenhouse gas research.

After the construction of a reservoir, there will be alterations in the hydrological condition, sediment deposition, and nutrient level of the river [6]. The generation of methane in reservoirs mainly relies on the production of methanogens in anaerobic sediments [7]. Methanogens make up a significant portion of the entire archaeal community, and as a result, the majority of global methane originates from their metabolism [8]. Based on different substrates, methanogens primarily produce methane through three pathways: (1) the hydrogenotrophic pathway, in which carbon dioxide and hydrogen react to produce methane and water (CO_2_ + H_2_→CH_4_ + H_2_O). (2) The acetoclastic pathway, in which acetic acid is decomposed into carbon dioxide and methane (CH_3_COOH→CO_2_ + CH_4_). (3) The methylotrophic pathway, in which methanol or other methyl compounds are converted to methane (CH_3_OH + CH_3_R→CH_4_) [9]. In most hypoxic environments, methane is generated through acetoclastic or hydrogenotrophic pathways [10]. The methylotrophic pathway is the predominant pathway for methane production in saline and marine environments [11], while in the sediment of freshwater ecosystems, the main pathways for methane production are hydrogenotrophic and acetoclastic pathways [10]. Hydrogenotrophic and acetoclastic methanogens account for 30% and 70% of methane production, respectively [12]. The dominant methanogenic communities for methane production mainly consist of *Methanoregula*, *Methanolinea*, *Methanobacterium*, and *Methanosaeta* [13].

With the development of global warming and humidification, more than half of the freshwater ecosystems in northern China are experiencing significant impacts. For example, occurrences such as increasing water temperature, decreasing dissolved oxygen concentration in water, and accelerated glacier melting will promote methane emissions [14]. In comparison to lakes, reservoirs often have a larger catchment area, which may result in the reservoir receiving more organic matter input from the surrounding catchment areas [15]. Consequently, this leads to reservoirs exhibiting a higher carbon burial rate and promoting methane production [16]. Currently, the majority of research focuses on the microbial community composition and metabolic functions of methanogens in freshwater lake sediments and paddy field soils. Studies on the Amazon and Pantanal lakes have shown that over 50% of CH_4_ production is attributed to hydrogenotrophic methanogens [17]. Seasonal temperature changes have a significant impact on the community composition and structure of microorganisms in paddy fields and wetland ecosystems [18]. However, the effects of season and basin differences on methanogenic communities have been largely overlooked [19,20]. Current research on methanogens in reservoirs has mainly focused on large rivers, without considering the potential impact of differences between reservoirs in large and small rivers. Although a few studies have investigated methane emissions from cascade reservoirs, these studies have primarily concentrated on the southwestern region of China [21,22], with relatively limited research on reservoirs in the Qinghai Plateau. The characteristics of methanogenic communities in sediments as well as their metabolic pathways remain unclear. However, the unique environmental conditions of the Qinghai Plateau, such as low temperature, low oxygen level, and intense ultraviolet radiation, may exert distinct influences on the methanogenic community. Consequently, these factors could potentially impact the ecological balance within reservoirs and greenhouse gas emissions.

Does the composition of methanogenic communities in the reservoir sediments on the northeastern Qinghai Plateau vary with season and basin? What are the metabolic pathways of methanogenic communities? Are there spatiotemporal differences? What are the main environmental factors influencing methanogenic communities? The answers to these questions are of great significance for the cause of energy conservation and emission reduction. However, the relevant scientific issues have not been deeply and systematically explained. Therefore, this study takes 18 reservoirs in the northeastern Qinghai Plateau as the research objects. It investigates the characteristics of methanogenic community structure, co-occurrence networks, and metabolic functions in reservoir sediments. This study also explores the main environmental factors affecting the characteristics of methanogenic communities in reservoir sediments with an aim to deepen our understanding of the mechanism of methane emissions from reservoirs on the Qinghai Plateau.

## 2. Materials and Methods

### 2.1. Sampling Site

**Figure 1:**
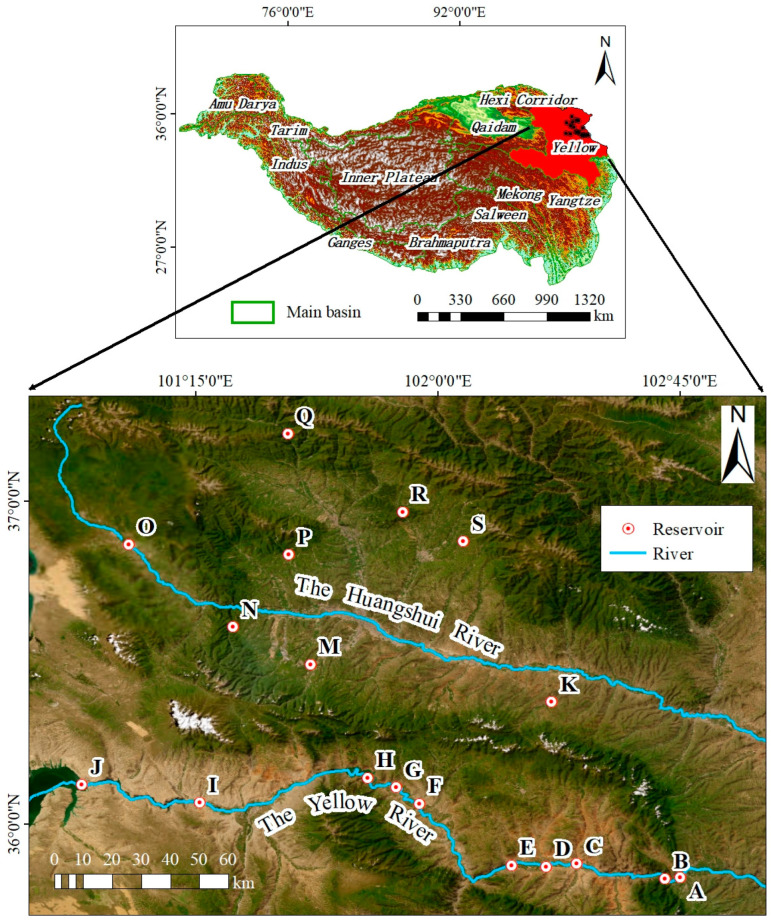
Distribution map of reservoirs. The letters on the map represent reservoirs with specific names listed in Section 2.1.

The research area (Figure 1) is located in the northeastern Qinghai Plateau (100°72′ E–102°75′ E, 35°83′ N–37°20′ N), encompassing 10 reservoirs on the main stream of the Yellow River (A: Dahejia, B: Jishixia, C: Huangfeng, D: Suzhi, E: Gongboxia, F: Kangyang, G: Zhiganglaka, H: Lijiaxia, I: Nina, and J: Longyangxia), as well as 8 reservoirs in the tributary (the Huangshui River) of the Yellow River (K: Shengjiaxia, M: Dananchuan, N: Pandao, O: Dongdatan, P: Yunguchuan, Q: Heiquan, R: Nanmenxia, and S: Zanzha). It is a transitional zone from the Loess Plateau to the Qinghai Plateau and also the boundary zone between China’s first- and second-order terrain. The area consists of low-altitude river valleys (elevation 1600–2700 m) interspersed with high-altitude mountains (elevation 3000–5000 m), with an overall terrain characterized by high in the west and low in the east. The climate type belongs to a typical plateau continental climate with an annual average temperature of about 5 ℃ and an annual average precipitation of about 360 mm. This area has many cliffs and wide valleys which are strongly affected by intense seismic activity, leading to frequent occurrences of landslides and debris flow. The soil types mainly include chestnut soil, cold desert soil, gray cinnamonic soil, etc., [23]. There are numerous deserts on both sides of the upper Yellow River with sand being dominant sediments in the floodplains. Frequent agricultural activities along the banks of the Huangshui River have a significant impact on the water quality of reservoirs and the physical and chemical properties of sediments [24]. The excessive construction of reservoirs in the northeastern Qinghai Plateau has to some extent altered the hydrological characteristics and microbial community structure of rivers, affecting natural ecological processes [25].

### 2.2. Sample Collection and Preservation

In May 2023 (dry season) and August 2023 (wet season), water samples and surface sediment samples were collected from the reservoirs. Each sampling was completed within 4–5 days. Based on field investigations, combined with the area of each reservoir and its distance from the dam, six parallel sampling points were set up in Longyangxia (J) and Lijiaxia (H), while three parallel sampling points were set up in the remaining sixteen reservoirs. Due to the closure of Gongboxia (E) in May, data for that month are missing. Additionally, due to road collapse in Lijiaxia (H) in August, sampling work could not be carried out, resulting in missing data for that month. The stainless-steel water sampler was positioned 0.5 m below the water surface to gather water samples, which were then stored in pre-sterilized polyethylene sampling bottles and maintained at 4 ℃ for the determination of water quality indicators. Sediment samples (approximately 10 cm of surface sediment) were obtained using a Petersen grab sampler. Following the mixing of the sediment samples, they were filtered through a 2 mm sieve, with a portion being placed in a 20 mL sterile centrifuge tube and stored in a liquid nitrogen tank for high-throughput sequencing of methanogens in the sediments. The remaining portion was placed in polyethylene sealable bags for the determination of physical and chemical properties of sediments. All samples were collected and stored according to relevant regulations outlined in Methods for Water and Wastewater Determination [26].

### 2.3. Environmental Data Collection and Determination

The sediment samples were freeze-dried for 24 h to remove moisture. The total organic carbon (sOC) content of the sediment samples was determined using the potassium dichromate oxidation-spectrophotometry method, as described by Luo et al. [27]. Total nitrogen (sTN) was measured using the Kjeldahl method, and total phosphorus (sTP) was determined using the alkaline fusion-molybdenum antimony anti-spectrophotometry method. The pH (spH) was measured using the potentiometric method. The water pH (wpH), total organic carbon (wTOC), total nitrogen (wTN), and total phosphorus (wTP) contents were determined according to national standards [26]. The water temperature (wT) and sediment temperature (sT) at each sampling point were measured using a YSI portable water quality detector, while GPS devices were used to record the elevation data of the sampling points. Data on maximum reservoir surface area came from https://doi.org/10.5281/zenodo.6984619, and precipitation data came from https://worldclim.org (22 June 2024).

### 2.4. DNA Extraction and PCR Amplification of Methanogens

The Powersoil DNA Isolation Kit was used to extract DNA from sediment samples, and the quality of the DNA was checked using 1% agarose gel electrophoresis. The qualified DNA was stored at −80 °C for further use. The ABI GeneAmp 9700 PCR system was employed to amplify the mcrA gene sequence of methanogens using the forward primer MLf (5′-GGTGGTGTMGGATTCACACARTAYGCWACAGC-3′) and reverse primer MLr (5′-TTCATTGCRTAGTTWGGRTAGTT-3′) [28]. To ensure accuracy, three replicates of PCR amplification were performed for each sample, and the products from the same sample were pooled after amplification. The PCR amplification parameters for methanogens were as follows: an initial denaturation at 95 °C for 3 min, followed by 30 cycles of denaturation at 95 °C for 30 s, annealing at 55 °C for 30 s, extension at 72 °C for 45 s, and a final extension at 72 °C for 10 min. The quality of PCR products was assessed by electrophoresis on 2% agarose gel, and qualified amplicons were subjected to high-throughput sequencing using an MiSeq PE300 sequencer (Illumina Inc., California, CA, USA).

### 2.5. Statistical Analysis

After conducting quality control, filtering, and splicing of the original sequence, clustering analysis was performed using the Usearch software (version 11) platform (USEARCH11-uparse algorithm) to obtain Operational Taxonomic Units (OTUs) at a 97% similarity level with a classification confidence of 0.7. All statistical analyses and visualizations were completed using R 4.3.2. The alpha diversity analysis was conducted and plotted using the vegan (version 2.6-4) and ggplot2 (version 3.5.1) package. Non-metric multidimensional scaling (NMDS) at the OTU level based on the Bray–Curtis distance was visualized using the vegan package, with further significance testing through permutational multivariate analysis of variance (PERMANOVA). Mantel analysis was used to explore environmental factors influencing the composition and diversity of methanogenic communities in the reservoir sediments on the northeastern Qinghai Plateau. The relative abundance of methanogenic communities at the genus level, co-occurrence network analysis, and functional prediction by FAPROTAX were analyzed and visualized using the Microeco package (version 1.6.1). Additionally, Gephi software (version 0.10.1) was used for the visualization of co-occurrence networks at the OTU level with a correlation coefficient greater than 0.6 and a *p*-value less than 0.05 [29].

## 3. Results

### 3.1. Composition of Methanogens

The high-throughput sequencing generated a total of 1,415,860 sequences of the mcrA gene from methanogenic archaea, with an average sequence length of 420 bp. OTUs were defined at a 97% sequence similarity, resulting in a total of 919 OTUs. The species annotation results revealed the presence of 6 phyla, 10 classes, 13 orders, 22 families, 35 genera, and 75 species. At the phylum level, the species included *Euryarchaeota* (85.04%) and unclassified groups (14.96%). At the order level, the species comprised *Methanosarcinales* (34.43%), *Methanobacteriales* (34.04%), and unclassified groups (18.96%).

**Figure 2:**
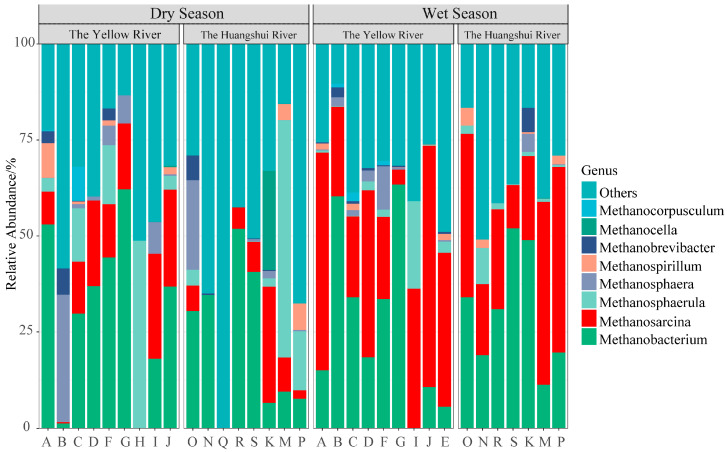
Community composition of methanogens in different seasons and basins. The capital letters represent the identification numbers of the reservoirs.

At the genus level (Figure 2), the relative abundance of methanogens at the genus level, with the top eight genera listed (the remaining grouped as “Other”), included *Methanobacterium* (28.87%), *Methanosarcina* (21.67%), *Methanosphaerula* (6.53%), *Methanosphaera* (3.24%), *Methanospirillum* (1.17%), *Methanobrevibacter* (0.98%), *Methanocella* (0.80%), and *Methanocorpusculum* (0.41%). Temporally, during the dry season, *Methanobacterium* was the dominant genus, while during the wet season, *Methanosarcina* took over as the dominant genus. Spatially, in the Yellow River basin, the dominant genus was *Methanosarcina*, whereas in the Huanshui River basin, it was dominated by *Methanobacterium*.

### 3.2. Diversity of Methanogens

**Figure 3:**
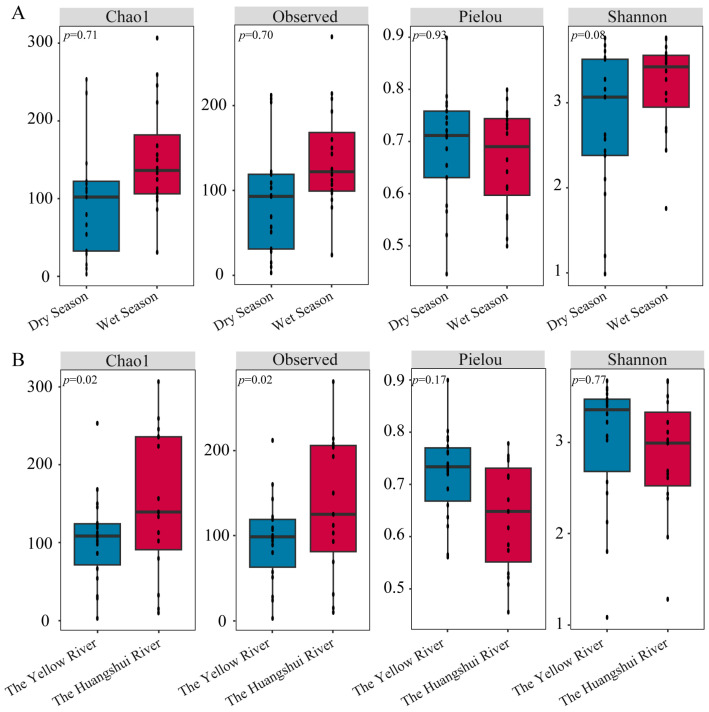
Alpha diversity of methanogens in different seasons (**A**) and basins (**B**).

The Chao1, Observed, Pielou, and Shannon indices were calculated using OTU data (Figure 3). Chao1 and Observed represent the number of OTUs, while Pielou and Shannon are primarily used to describe the diversity of community. Higher Shannon and Pielou indices indicate greater species diversity. When considering different seasons (Figure 3A), the Chao1 and Observed indices during the wet season were higher than those during the dry season, but the difference was not significant (*p* > 0.05). The Pielou evenness and Shannon diversity index also showed higher values during the wet season compared to the dry season (*p* > 0.05). Comparing α-diversity among different river basins (Figure 3B), both Chao1 and Observed indices in the Yellow River basin were significantly higher than those in the Huangshui River basin (*p* < 0.05). However, the Pielou and Shannon indices in the Yellow River basin were higher than those in the Huangshui River basin (*p* > 0.05). Overall, differences in α-diversity of methanogenic communities in reservoir sediments on the northeastern Qinghai Plateau were greater among different river basins than among different seasons. These data for individual sites are shown in Table S1.

**Figure 4:**
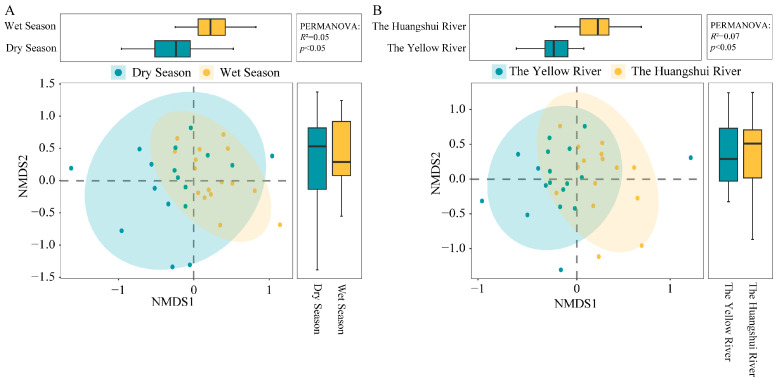
Analysis of methanogens using PERMANOVA and NMDS in different seasons (**A**) and basins (**B**).

To further demonstrate the diversity of species in the samples, non-metric multidimensional scaling (NMDS) based on the Bray–Curtis distance at the OTU level was used to show the differences between different samples. The NMDS ordination plot showed an overlap in the methanogenic communities between different seasons and river basins. There is a larger overlap between methanogenic communities in different seasons, indicating a higher similarity, with the most densely distributed methanogenic community during the wet season (Figure 4). Permutational multivariate analysis of variance (PERMANOVA) based on distance matrices revealed significant differences in β-diversity of methanogenic communities among different seasons and river basins in sediment samples from reservoirs on the northeastern Qinghai Plateau (*p* < 0.05) (Figure 4A,B).

### 3.3. Co-Occurrence Network of Methanogens

**Figure 5:**
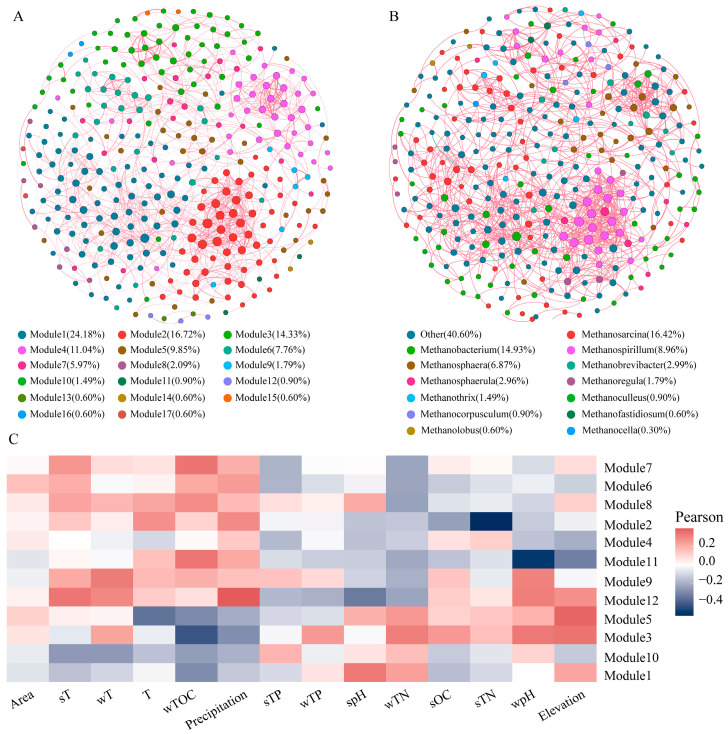
Co-occurrence network of methanogenic community constructed based on the spearman correlation matrix at the OTU level. (**A**) Nodes are colored by module. (**B**) Nodes are colored by methanogen genus. (**C**) Correlation between modules and environmental factors.

Based on the Spearman correlation (*r* > 0.6, *p* < 0.05), a co-occurrence network of methanogenic communities in the sediments was constructed (Figure 5). The network consisted of 335 nodes and 1140 edges. The number of positive correlations (99.82%) far exceeds that of negative correlations (0.18%), indicating that cooperation was the main function. The network demonstrated a robust modular structure, with a modularity index of 0.69 (exceeding the threshold of 0.44). Furthermore, the topological parameters were as follows: an average degree of 6.81, an average clustering coefficient of 0.43, an average path length of 5.41, a network diameter of 16, and an average weighted degree of 4.84. Overall, there was good clustering among nodes in the methanogenic network in reservoir sediments, with high connectivity between OTUs. When the nodes in the network were divided by genus (Figure 5B), it was found that *Methanosarcina* (16.42%) and *Methanobacterium* (14.93%) had relatively high proportions among all nodes. Unannotated taxa accounted for 40.60% of total nodes, while other genera had smaller proportions. Furthermore, when nodes were divided into modules within the co-occurrence network for methanogens in sediments (Figure 5A), it was discovered that there were seventeen modules present within this network. However, twelve out of these seventeen modules accounted for approximately 97%.

Figure 5C illustrates the influence of environmental variables on the modules of methanogens in the co-occurrence network. Modules 1 and 10 show negative correlations with Area, sT, wT, T, wTOC, and Precipitation, while modules 2, 6, 7, 8, and 12 exhibit positive correlations with these variables. Additionally, modules 3 and 5 are positively correlated with wTN, sOC, sTN, wpH, and elevation, whereas modules 6 and 11 are negatively correlated with these variables. Module 8 demonstrates positive correlations with most environmental parameters while module 4 shows negative correlations. Furthermore, module 9 is positively associated with sT, wT, T, wTOC, Precipitation, sTP, wTP, sOC, and wpH, but negatively related to Area, spH, wTN, sTN, and elevation.

### 3.4. FAPROTAX Ecological Function Prediction of Methanogens

**Figure 6:**
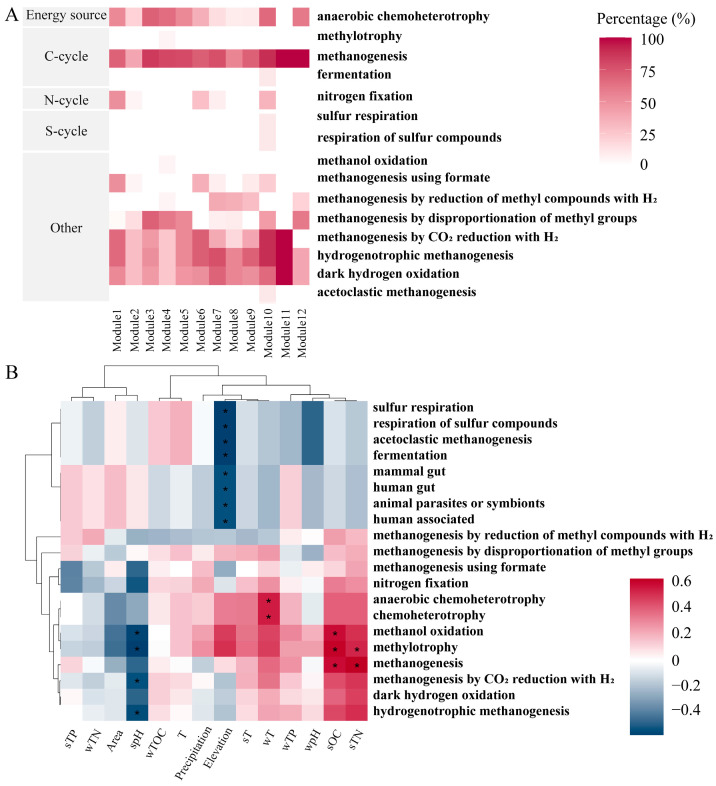
FAPROTAX function prediction of methanogens. (**A**) Methanogenic active groups. (**B**) Correlation of environmental factors and active groups. The asterisk indicates significance at a *p* < 0.05 level.

The FAPROTAX database was used to annotate the co-occurrence network modules of the methanogenic community in the reservoir sediments, resulting in a total of 15 active groups (Figure 6A). These mainly included aerobic chemoheterotrophy, methanogenesis, methanogenesis using formate, methanogenesis by the reduction of methyl compounds with H_2_, methanogenesis by the disproportionation of methyl groups, methanogenesis by CO_2_ reduction with H_2_, hydrogenotrophic methanogenesis, and dark hydrogen oxidation. The above active groups accounted for more than 50% of all measured functional groups, indicating that over half of the metabolic types of methanogens in the sediments may be hydrogenotrophic pathways using CO_2_/H_2_ as substrates. This was because there was little difference in the metabolic pathways between methanogenic communities in different basins and seasons (Figure S1), with the hydrogenotrophic pathway being dominant. 

Analysis of the correlation between environmental factors and functional groups showed that methanogenesis and methylotrophy were significantly positively correlated with sOC and sTN, respectively (*p* < 0.05) (Figure 6B). Methanol oxidation was found to be positively correlated with sOC (*p* < 0.05), but negatively correlated with spH (*p* < 0.05). Additionally, anaerobic chemoheterotrophy and chemoheterotrophy were significantly positively correlated with wT (*p* < 0.05). Conversely, sulfur respiration, the respiration of sulfur compounds, fermentation, the mammal gut, the human gut, animal parasites or symbionts, and human-associated were significantly negatively correlated with altitude (*p* < 0.05). Furthermore, spH was negatively correlated with methylotrophy, methanogenesis by CO_2_ reduction with H_2_, and hydrogenotrophic methanogenesis (*p* < 0.05). The results indicated that spH, elevation, wT, sOC, and sTN played a major role in regulating the function of methanogens deposited in reservoirs on the northeastern Qinghai Plateau.

## 4. Discussion

### 4.1. Temporal and Spatial Characteristics of Methanogenic Composition and Diversity

The composition of methanogenic communities in the surface sediment of the reservoir on the northeastern Qinghai Plateau revealed *Methanobacterium* and *Methanosarcina* as dominant genera, which is consistent with previous studies [30,31]. The composition of methanogenic communities varied spatiotemporally, likely due to differences in environmental factors and hydrological conditions. Differences in physicochemical parameters such as water temperature and dissolved oxygen between dry and wet seasons have been confirmed to be important influencing factors for microbial community [32,33]. *Methanosarcina* was a predominant genus in the Yellow River, exhibiting good environmental adaptability and playing a significant role in carbon cycling within reservoirs [34]. In freshwater sediment ecosystems, dominant methanogens are typically acetoclastic and hydrogenotrophic methanogens, such as *Methanoregula* [35], consistent with the findings of this study. However, Ren et al. [36] reported different predominant species (*Methanosaetaceae* and *Methanomicrobiales*) in the sediments from Lake Gongzhucuo and Lake Zige Tangcou compared to our results. This difference may be related to temperature. These lakes have an average annual temperature ranging from 0 to 2.5 ℃, where acetoclastic or hydrogenotrophic methanogens were dominant species even at low temperatures. In contrast, the sampled reservoir had an average annual temperature ranging from 4 to 8 ℃, with predominantly hydrogenotrophic methanogens as the dominant community.

In this study, it was found that the Chao1, Observed, Pielou, and Shannon indexes of methanogens in the sediments were higher during the wet season than during the dry season. This indicated that the methanogens during the wet season were more abundant and had a more complex community structure, which is consistent with previous research conclusions [37,38]. According to general survival theory, under more extreme (cold) environmental conditions, the number of microorganisms in lakes and reservoirs was usually lower and community structures tended to be simpler [39]. Low temperatures can lead to the formation of ice crystals in the cytoplasm, directly damaging cells and causing an imbalance in the internal osmotic pressure [40]. In terms of spatial distribution, the species abundance of methanogens in the Huangshui River basin was richer than that in the Yellow River basin (*p* < 0.05). This may be related to the relatively closed and slow flow characteristics in small reservoirs. These environmental conditions were favorable for the growth and reproduction of methanogens. In contrast, large reservoirs with faster flow and frequent water exchange may not be conducive to the aggregation and growth of methanogens [30]. Additionally, methanogens are strict anaerobes that can thrive better under anaerobic environments [41]. The greater richness of methanogenic communities in the Yellow River basin compared to the Huangshui River basin suggested a possible association with a more complex ecological environment typically found in large reservoirs. The diversity within these ecological environments provides suitable habitats for different types of methanogens [42]. Furthermore, large reservoirs in major river basins generally have larger water surface areas and greater water depths, providing a broader habitat for methanogens [43].

### 4.2. Influencing Factors on the Composition and Diversity of Methanogens

**Figure 7:**
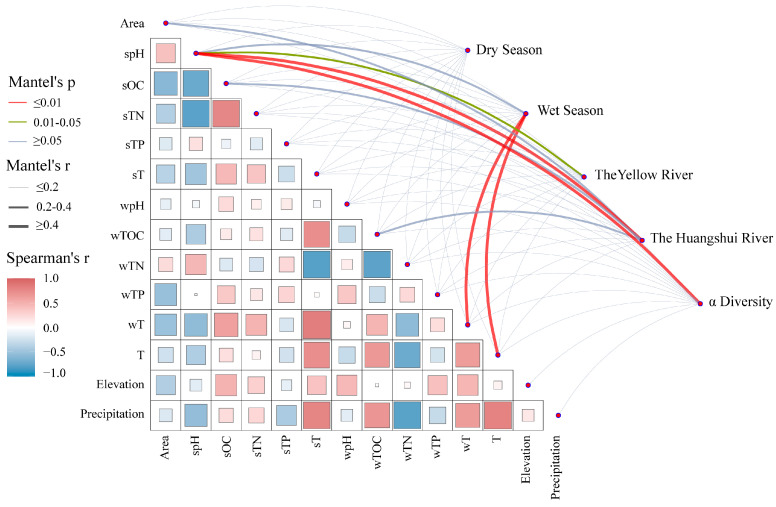
Relationship between methanogenic community and α diversity and environmental factors. The size of the boxes in the lower left panel represents the magnitude of the correlation between environmental factors, while the color indicates whether the correlation is positive or negative. The upper right panel shows the relationship between community composition and methanogenic diversity with environmental factors across different seasons and basins. The color of the lines represents the significance level, and the thickness indicates the strength of correlation with environmental factors.

The characteristics of microbial communities are closely related to the environment [44]. Previous studies have shown that organic matter, water temperature, and pH are important factors influencing the composition and structure of microbial communities in freshwater ecosystems [45,46]. We conducted Mantel analysis to investigate the relationship between environmental factors and the composition and diversity of methanogenic communities (specific environmental parameters can be found in Tables S2 and S3). The results (Figure 7) show that, seasonally, environmental factors did not significantly affect the composition of methanogenic communities during the dry season (*p* > 0.05), while during the wet season, there was a significant correlation between the composition of methanogenic communities and wT (*r* = 0.48, *p* < 0.01) as well as T (*r* = 0.45, *p* < 0.01). In different river basins, there was a significant correlation between the composition of methanogenic communities in the Yellow River basin and spH (*r* = 0.28, *p* < 0.05), while in the Huangshui River basin, there was an extremely significant correlation with spH (*r* = 0.43, *p* < 0.01). The alpha diversity of methanogens in reservoir sediments showed an extremely significant correlation with spH (*r* = 0.46, *p* < 0.01). Overall, wT, T, and spH significantly influenced both the community composition and alpha diversity of methanogens in the reservoir sediments on the northeastern Qinghai Plateau (*p* < 0.05), which is consistent with previous research results [47,48]. Related studies have shown that temperature is an important environmental parameter in aquatic ecosystems and can have a significant impact on the metabolic activity and community structure of methanogenic microorganisms [49,50]. Temperature mainly affects the process of methane production by regulating the metabolic rate of microorganisms, and changes in temperature can affect the enzyme activity within microbial cells. Based on different optimal growth temperatures, methanogens can be divided into four groups: psychrophiles (optimal temperature below 25 °C), mesophiles (optimal temperature around 35 °C), thermophiles (optimal temperature around 55 °C), and hyperthermophiles (optimal temperature above 80 °C) [51]. For example, *acetoclastic Methanosaeta* dominates under low-temperature conditions [52], while *Methanogenium* cannot survive in places with temperatures above 20 °C [53]. Additionally, methanogens are sensitive to changes in pH [54]. In lakes with high salinity and pH, there is a significant increase in the abundance of hydrogenotrophic methanogens [9]. Research has also shown that adding different pH buffer solutions to acetic-acid-producing methanogens significantly affects methane production trends and microbial community structure [55]. Changes in pH may also indirectly affect the composition of methanogenic communities by influencing the activity of other microorganisms [56].

### 4.3. Hydrogenotrophic Methanogenesis Was the Dominant Pathway

The metabolic pathway of methanogens involves the production of CH_4_ using acetic acid, CO_2_, and some simple methyl compounds as substrates [54]. This process mainly includes three pathways to generate methane: hydrogenotrophic pathway, acetoclastic pathway, and methylotrophic pathway. All three pathways ultimately form methyl-coenzyme M, which is then catalyzed by methyl-coenzyme M reductase to produce CH_4_ [50]. Due to the low content of methylated compounds in freshwater ecosystems, acetoclastic and hydrogenotrophic pathways are the main CH_4_ production pathways in freshwater ecosystems [49]. The dominant methanogens in the surface sediments of reservoirs in the northeastern Qinghai Plateau were *Methanosarcina* and *Methanobacterium*. *Methanobacterium* belongs to the hydrogenotrophic methanogens, which can convert CO_2_ and H_2_ into CH_4_ [57]. *Methanosarcina* has a flexible substrate metabolism and can utilize H_2_, methanol, methylamine, and acetic acid for growth. It is an amphitropic methanogen [58]. It is inferred that the hydrogenotrophic pathway (H_2_/CO_2_ reduction) dominates methane production in the reservoir surface sediments on the northeastern Qinghai Plateau. The hydrogenotrophic pathway primarily uses H_2_ as an electron donor to reduce CO_2_ to generate CH_4_. Many methanogens can utilize this pathway [59]. In different basins and seasons, the metabolic pathways of methanogenic communities in reservoirs were mainly dominated by the hydrogenotrophic pathway. This may be related to the availability of substrates. Despite variations in environmental conditions across different river basins and months, CO_2_ and H_2_ as primary substrates for hydrogenotrophic pathway are widely available in aquatic environments. These two substrates provide a stable habitat for these organisms [49]. This dominance may also be related to energy advantages obtained from using the hydrogenotrophic pathway. This pathway has a higher energy output efficiency. Therefore, under different environmental conditions, hydrogenotrophic methanogens can effectively obtain energy through this pathway to maintain their life activities and metabolic needs [54].

## 5. Conclusions

With the continuous development of global climate change, significant alterations have occurred in the microbial basis of greenhouse gas emissions in the reservoir sediments on the Qinghai Plateau, characterized by warming and humidification. In this study, 18 reservoirs on the Qinghai Plateau were selected as research objects to explore the characteristics of methanogenic communities in sediments during the dry season (May 2023) and wet season (August 2023) using mcrA sequencing technology, FAPROTAX, and Mantel analysis. The main conclusions are as follows:

(1) The dominant genus of methanogens in the reservoir sediments on the northeastern Qinghai Plateau are *Methanobacterium* (28.87%) and *Methanosarcina* (21.67%). The hydrogenotrophic pathway using CO_2_/H_2_ as substrates predominates in the production of methane in reservoir sediments.
(2) The α-diversity of methanogenic communities is higher during the wet season compared to the dry season (*p* > 0.05). Additionally, the diversity of methanogens is greater in large reservoirs than in small reservoirs (*p* < 0.05). β-diversity exhibits significant differences between different seasons and river basins (*p* < 0.05). Furthermore, temperature and pH have a significant impact on the composition and diversity of methanogenic communities in the reservoir sediments on the northeastern Qinghai Plateau (*p* < 0.05).
(3) The network stability of methanogens in the reservoir sediments on the northeastern Qinghai Plateau is robust, as indicated by a modularity index of 0.69. The main function within this network is cooperation, with a high positive correlation edge number of 99.82%.

## Data Availability

The data presented in the study are available in this article and the original sequence data are stored in the NCBI database (PRJNA1126413).

## Supplementary Materials

The following supporting information can be downloaded at: https://www.mdpi.com/article/10.3390/biology13080615/s1, Table S1: Alpha diversity data of each site; Table S2: Specific environmental parameters (Dry season). Table S3: Specific environmental parameters (Wet season). Figure S1: FAPROTAX function prediction of methanogens. (A: Dry season; B: Wet season; C: The Yellow River; D: The Huangshui River).
