# Supplementary material for: Methanogenic Community Characteristics and Its Influencing Factors in Reservoir Sediments on the Northeastern Qinghai Plateau
Source: Biology (Basel). 2024 Aug 14;13(8):615. doi: 10.3390/biology13080615 (PMC11351834; doi:10.3390/biology13080615)
Supplement: Supplementary file 1 [file biology-13-00615-s001.zip › biology-3129339-supplementary.pdf]

Table S1: Alpha diversity data of each site.

ID	Chao1		Observed		Pielou		Shannon	
	Dry Season	Wet Season	Dry Season	Wet Season	Dry Season	Wet Season	Dry Season	Wet Season
A	141.00	165.50	122.00	148.00	0.74	0.71	3.53	3.55
B	125.33	108.33	95.00	99.00	0.72	0.73	3.28	3.36
C	133.60	130.25	118.00	119.00	0.78	0.73	3.73	3.47
D	54.00	102.00	51.00	97.00	0.66	0.79	2.58	3.63
E	-	169.15	-	156.00	-	0.61	-	3.09
F	112.63	97.50	107.00	90.00	0.77	0.78	3.61	3.51
G	65.50	91.14	55.00	86.00	0.76	0.55	3.06	2.44
H	3.00	-	3.00	-	0.90	-	0.99	-
I	32.75	27.50	29.00	26.00	0.62	0.55	2.09	1.78
J	233.14	119.00	208.00	108.00	0.68	0.75	3.65	3.53
K	33.50	136.43	32.00	127.00	0.71	0.74	2.45	3.59
M	237.15	127.36	213.00	115.00	0.45	0.64	2.41	3.04
N	15.33	318.57	15.00	286.00	0.71	0.66	1.93	3.72
O	106.67	214.04	98.00	192.00	0.77	0.51	3.51	2.69
P	251.39	262.13	215.00	221.00	0.57	0.61	3.09	3.28
Q	10.00	-	10.00	-	0.52	-	1.19	-
R	74.75	165.60	71.00	150.00	0.74	0.75	3.16	3.77
S	114.40	233.41	103.00	209.00	0.57	0.50	2.65	2.66

Table S2: Specific environmental parameters (Dry season).

ID	Area (km^2)	spH	sOC (g/kg)	sTN (g/kg)	sTP (g/kg)	sT (℃)	wpH	wTOC (mg/L)	wTN (mg/L)	wTP (mg/L)	wT (℃)	T (℃)	Precipitation (mm)
A	1.00	8.75	2.21	1.56	0.37	12.40		21.78	2.67	0.13	13.07	22.90	52.00
B	12.86	9.17	2.61	1.34	0.26	9.80	8.55	8.49	5.93	0.02	9.77	12.80	51.00
C	5.04	8.88	5.87	1.89	0.32	9.67	8.52	4.73	6.71	0.02	8.63	13.50	50.00
D	6.53	8.80	5.22	1.77	0.34	9.20	8.53	1.30	7.06	0.02	8.60	14.03	51.00
E	-	-	-	-	-	-	8.58	-	-	-	-	-	-
F	5.59	8.00	11.31	2.48	0.29	7.70	-	3.01	7.12	0.02	7.83	10.30	48.00
G	1.61	8.65	2.98	1.78	0.43	7.70	8.66	7.06	5.77	0.02	6.87	14.37	48.00
H	36.25	9.45	2.50	1.67	0.47	11.98	8.69	15.75	3.73	0.01	12.10	14.43	49.00
I	2.54	9.34	2.01	1.56	0.50	7.30	8.68	24.71	7.17	0.01	6.20	9.10	49.00
J	413.77	8.63	6.49	1.76	0.33	8.75	8.71	21.11	7.03	0.02	9.05	9.47	47.00
K	0.29	8.47	9.85	1.88	0.38	14.80	8.70	21.68	4.96	0.03	13.63	17.03	48.00
M	0.61	8.19	28.42	7.17	0.23	14.80	8.43	38.89	4.67	0.03	15.27	14.07	51.00
N	0.97	8.86	5.83	1.45	0.43	15.50	8.57	29.55	5.16	0.02	15.17	25.23	54.00
O	3.19	8.29	6.02	2.34	0.32	11.98	8.74	29.39	8.28	0.03	11.63	11.53	51.00

P	0.41	7.46	27.70	4.29	0.38	12.40	8.71	17.74	2.46	0.03	14.27	13.07	58.00
Q	5.01	9.27	2.69	1.65	0.28	12.35	8.87	7.92	6.01	0.03	12.30	22.40	58.00
R	1.11	8.71	9.84	1.79	0.38	15.10	8.53	17.59	5.96	0.02	14.50	19.70	53.00
S	0.18	8.45	10.23	2.13	0.42	18.20	8.49	30.20	4.57	0.03	19.30	13.50	50.00

Table S3: Specific environmental parameters (Wet season).

ID	spH	sOC (g/kg)	sTN (g/kg)	sTP (g/kg)	sT (℃)	wpH	wTOC (mg/L)	wTN (mg/L)	wTP (mg/L)	wT (℃)	T (℃)	Elevation (m)	Precipitation (mm)
A	8.06	4.85	2.04	0.43	14.10	7.95	48.56	0.92	0.01	14.95	25.33	1756.44	87.00
B	8.18	5.59	2.08	0.38	17.48	8.15	52.14	1.01	0.01	13.80	29.60	1811.97	86.00
C	8.28	4.54	1.87	0.24	17.57	8.13	56.06	0.97	0.01	14.47	25.92	1822.95	83.00
D	8.30	4.56	1.66	0.22	14.10	8.43	34.10	1.01	0.01	13.73	29.15	1850.89	83.00
E	8.32	4.19	1.80	0.27	20.80	8.48	49.02	0.33	0.01	12.67	22.60	2005.00	92.00
F	8.39	6.01	1.73	0.38	17.30	8.41	46.68	1.12	0.01	13.30	29.80	1993.76	78.00
G	8.41	5.26	1.70	0.24	12.40	8.35	48.66	0.88	0.01	9.10	32.33	2002.45	78.00
H	-	-	-	-	-	-	-	-	-	-	-	-	-
I	8.77	3.33	1.71	0.29	18.58	8.56	54.08	0.66	0.01	7.27	16.27	2190.67	76.00
J	8.75	4.72	1.97	0.09	20.67	8.68	40.04	0.86	0.01	19.68	20.25	2532.99	69.00
K	8.20	15.81	1.87	0.41	22.30	8.92	49.50	0.29	0.03	23.63	28.87	2260.00	83.00
M	7.82	38.09	4.64	0.59	27.70	8.70	50.70	0.38	0.03	21.77	32.63	2585.00	85.00
N	8.10	18.07	1.97	0.22	25.80	8.59	42.37	1.02	0.02	25.10	33.83	2780.00	89.00
O	8.12	7.15	1.91	0.28	25.60	8.80	35.74	0.98	0.02	21.87	26.50	2938.00	90.00
P	7.86	17.42	2.40	0.18	19.80	8.91	21.79	0.39	0.01	18.60	24.23	2696.00	95.00
Q	-	-	-	-	-	-	-	-	-	-	-	-	-
R	8.27	9.31	2.13	0.15	20.80	8.48	41.72	1.16	0.01	20.63	30.03	2757.00	94.00
S	8.24	11.33	2.34	0.25	20.80	8.51	40.51	0.86	0.03	20.87	33.23	2724.00	91.00

Figure **S1: FAPROTAX function prediction of methanogens. (A: Dry season; B: Wet season; C: The Yellow River; D: The Huangshui River)**

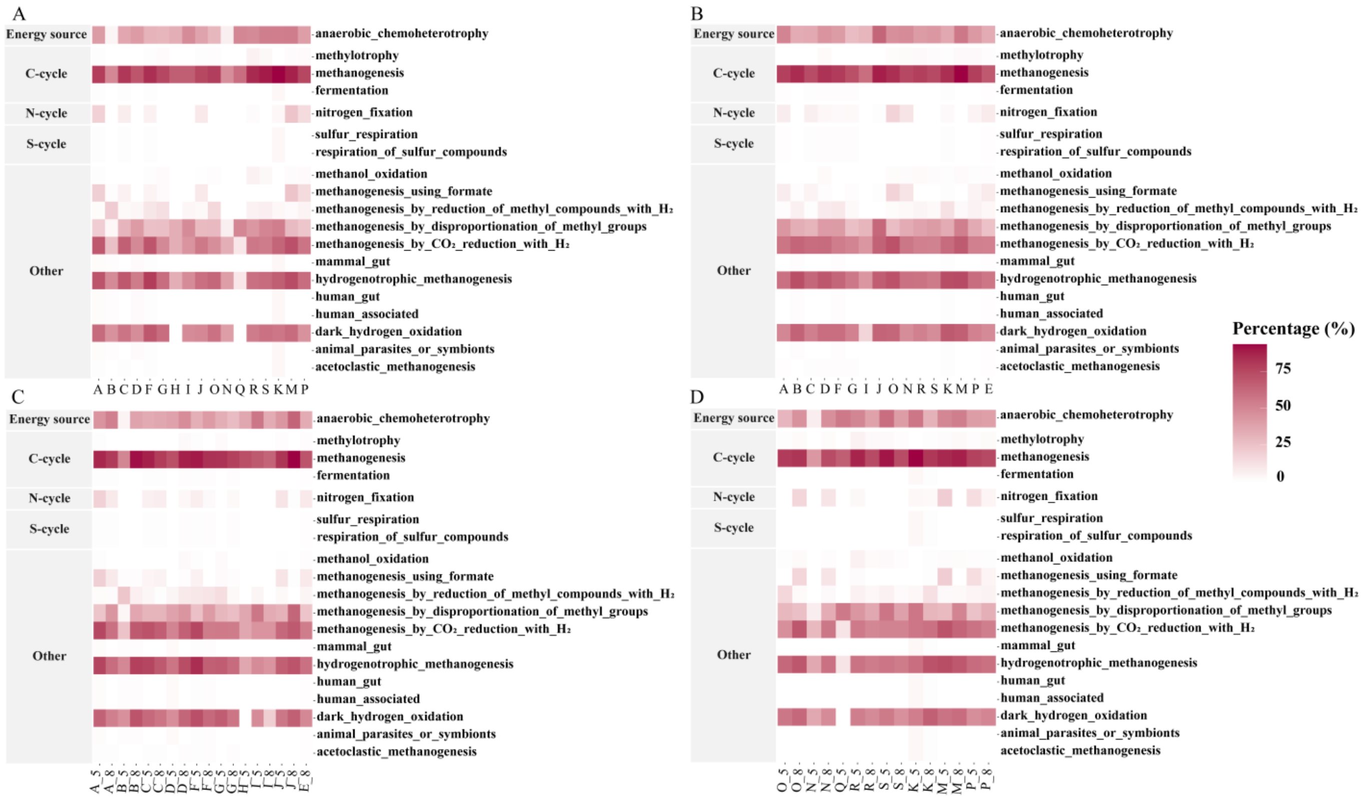

A
Energy source
C-cycle
N-cycle
S-cycle
Other
anaerobic_chemoheterotrophy
methylotrophy
methanogenesis
fermentation
nitrogen_fixation
sulfur_respiration
respiration_of_sulfur_compounds
methanol_oxidation
methanogenesis_using_formate
methanogenesis_by_reduction_of_methyl_compounds_with_H2
methanogenesis_by_disproportionation_of_methyl_groups
methanogenesis_by_CO2_reduction_with_H2
mammal_gut
hydrogenotrophic_methanogenesis
human_gut
human_associated
dark_hydrogen_oxidation
animal_parasites_or_symbionts
acetoclastic_methanogenesis
A B C D F G H I J O N Q R S K M P
B
Energy source
C-cycle
N-cycle
S-cycle
Other
anaerobic_chemoheterotrophy
methylotrophy
methanogenesis
fermentation
nitrogen_fixation
sulfur_respiration
respiration_of_sulfur_compounds
methanol_oxidation
methanogenesis_using_formate
methanogenesis_by_reduction_of_methyl_compounds_with_H2
methanogenesis_by_disproportionation_of_methyl_groups
methanogenesis_by_CO2_reduction_with_H2
mammal_gut
hydrogenotrophic_methanogenesis
human_gut
human_associated
dark_hydrogen_oxidation
animal_parasites_or_symbionts
acetoclastic_methanogenesis
A B C D F G I J O N R S K M P E
Percentage (%)
75
50
25
0
C
Energy source
C-cycle
N-cycle
S-cycle
Other
anaerobic_chemoheterotrophy
methylotrophy
methanogenesis
fermentation
nitrogen_fixation
sulfur_respiration
respiration_of_sulfur_compounds
methanol_oxidation
methanogenesis_using_formate
methanogenesis_by_reduction_of_methyl_compounds_with_H2
methanogenesis_by_disproportionation_of_methyl_groups
methanogenesis_by_CO2_reduction_with_H2
mammal_gut
hydrogenotrophic_methanogenesis
human_gut
human_associated
dark_hydrogen_oxidation
animal_parasites_or_symbionts
acetoclastic_methanogenesis
A_5 A_8 B_5 B_8 C_5 C_8 D_5 D_8 F_5 F_8 G_5 G_8 H_5 I_5 I_8 J_5 J_8 E_8
D
Energy source
C-cycle
N-cycle
S-cycle
Other
anaerobic_chemoheterotrophy
methylotrophy
methanogenesis
fermentation
nitrogen_fixation
sulfur_respiration
respiration_of_sulfur_compounds
methanol_oxidation
methanogenesis_using_formate
methanogenesis_by_reduction_of_methyl_compounds_with_H2
methanogenesis_by_disproportionation_of_methyl_groups
methanogenesis_by_CO2_reduction_with_H2
mammal_gut
hydrogenotrophic_methanogenesis
human_gut
human_associated
dark_hydrogen_oxidation
animal_parasites_or_symbionts
acetoclastic_methanogenesis
O_5 O_8 N_5 N_8 Q_5 R_5 R_8 S_5 S_8 K_5 K_8 M_5 M_8 P_5 P_8
